# The cost-effectiveness analysis of maintenance olaparib plus Bevacizumab in patients with advanced ovarian cancer: based on the final survival results from PAOLA-1 trial

**DOI:** 10.1186/s13048-023-01257-4

**Published:** 2023-08-21

**Authors:** Youwen Zhu, Kun Liu, Hui Cao, Hong Zhu

**Affiliations:** 1 Department of Oncology, Xiangya Hospital, Central South University, Changsha, Hunan 410008 China; 2 Department of Oncology, Chenzhou First People’s Hospital, Chenzhou, Hunan 423000 China; 3 National Clinical Research Center for Geriatric Disorders, Xiangya Hospital, Central South University, Changsha, Hunan 410008 China

**Keywords:** Advanced ovarian cancer, Olaparib plus bevacizumab, BRCA mutation, HRD-positive, Quality-adjusted life-years

## Abstract

**Introduction:**

In 2023, the final PAOLA-1 trial (NCT02477644) survival data were published documenting the benefits of therapy consisting of olaparib plus bevacizumab for patients with advanced ovarian cancer (AOC) as a function of molecular status. In light of these new data, the present study was conducted with the goal of evaluating the cost-effectiveness of olaparib plus bevacizumab for the treatment of the overall AOC patient population and for homologous recombination deficiency (HRD)-positive patients, patients with a breast cancer susceptibility gene (BRCA) mutations, homologous recombination proficiency (HRD)-positive, or patients not harboring BRCA mutations AOC from a US payers perspective.

**Methods:**

A Markov state-transition model with a 15-year time horizon was used to evaluate outcomes of patients administered Olaparib plus bevacizumab versus bevacizumab. Life-years (LYs), quality-adjusted LYs (QALYs), and the incremental cost-effectiveness ratio (ICER) values were evaluated in this study in light of a $150,000/QALY of willingness-to-pay (WTP) threshold. The stability of the established model was evaluated through sensitivity analyses.

**Results:**

Relative to bevacizumab alone, Olaparib plus bevacizumab was associated with mean incremental costs and QALYs (LYs) of olaparib plus bevacizumab versus bevacizumab were $293,656 and 1.85 (2.16), $265,668 and 3.34 (4.02), $242,746 and 1.71 (2.06), and $193,792 and 0.97 (1.14) for overall, BRCA mutation-positive, HRD-positive, and HRD-positive BRCA mutation-negative AOC patients, respectively. The corresponding ICER values for these patient subgroups were $158,729 ($136,218), $79,434 ($66,120), $141,636 ($117,747), and $200,595 ($169,733) per QALY (LY) gained Utility value and the price of olaparib were identified in sensitivity analyses as the primary factors influencing these results.

**Conclusion:**

At current pricing levels, maintenance treatment with olaparib plus bevacizumab treatment may represent a cost-effective therapeutic option for BRCA mutations and HRD-positive AOC patients in the USA.

**Supplementary Information:**

The online version contains supplementary material available at 10.1186/s13048-023-01257-4.

## Introduction

Ovarian cancer (OC) is the 11th leading malignancy among women in the USA and 5th deadliest, with 19,710 new diagnoses and 13,270 deaths forecast in 2023 alone [1]. Homologous recombination deficit (HRD) owing to the inactivation of resulting from breast cancer susceptibility genes 1 and 2 (BRCA1 and BRCA2) inactivation is a leading driver of oncogenic in individuals diagnosed with OC as a consequence of the impaired repair of double-stranded DNA [2]. BRCA gene mutations women face a higher risk of OC incidence, as these mutations have been linked to the greatest potential for [3, 4]. The most common OC tumors are those of epithelial origin, accounting for 70% of cases that are diagnosed in an advanced stage such that patients generally face poor prognostic outcomes and a 5-year survival rate of less than 30%[3, 5].

In newly diagnosed advanced OC (AOC) patients over the last decade has been surgical tumor cytoreduction with subsequent platinum and nonplatinum (taxane-based) drugs treatment has been the standard first-line therapeutic approach for more than 10 years [6]. However, In the large-scale phase III ICON7 (ISRCTN91273375) and GOG-0218 (NCT00262847) trials revealed that the median progression-free survival (PFS) of AOC patients that underwent this combination of surgery and chemotherapy was just 10–17 months, with themajority of these individuals ultimately developing recurrent disease [7, 8]. There is thus a clear need for innovative drugs or therapeutic strategies that can provide AOC patients with significant clinical benefits irrespective of their surgical or molecular status.

The monoclonal anti-vascular endothelial growth factor A (anti-VEGF-A) antibody bevacizumab has recently been developed and approved for therapeutic use. AOC patients administered a combination of bevacizumab plus chemotherapy with subsequent bevacizumab maintenance therapy attain significant clinical benefits irrespective of the disease stage and an apparent absence of post-surgical disease progression such that this regimen has emerged as the preferred option for the management of newly-diagnosed individuals with AOC [7–12]. Roughly half of all AOC patients present with tumors that are HRD-positive, with the majority of these being driven by mutations in BRCA genes [13]. This observation has spurred increased interest in selecting the most appropriate treatments for individuals diagnosed with AOC in particular molecular subgroups by detecting specific disease-associated biomarkers disease-associated biomarkers of interest. Olaparib is an oral poly (ADP-ribose) polymerase (PARP) inhibitor that has demonstrated value as a first-line option for treating AOC [14]. The presentation of final overall survival (OS) outcome data from the phase III PAOLA-1 trial (NCT02477644) at ESMO in 2023 revealed that relative to bevacizumab alone, olaparib plus bevacizumab significantly prolonged the median OS of AOC patients with HRD-positive tumors (75.2 months vs. 57.3 months; hazard ratio (HR), 0.62; 95% confidence interval (CI), 0.45 to 0.85) and BRCA mutation tumors (75.2 vs. 66.9; HR, 0.60; 95% CI, 0.39 to 0.93)[15]. This combination regimen, however, failed to provide significant benefits in the overall AOC patient cohort (56.5 vs. 51.6 months; HR, 0.92; 95% CI, 0.76 to 1.12) or in patients with HRD-positive tumors negative for BRCA mutations (NR vs. 52; HR, 0.71; 95% CI, 0.45 to 1.13)[15]. In light of these promising results, the analysis was updated this 5-year PFS data for this trial were presented at the 2023 ESMO meeting, revealing that olaparib plus bevacizumab yielded significant median PFS benefits over those associated with bevacizumab alone in the overall AOC patient population (22.9 vs. 16.6 months; HR, 0.63; 95% CI, 0.53 to 0.74), HRD-positive patients (46.8 vs. 17.6 months; HR, 0.41; 95% CI, 0.32 to 0.54), BRCA mutations patients (60.7 vs. 21.7 months; HR, 0.45; 95% CI, 0.32 to 0.64), and HRD-positive BRCA mutation-negative patients (30.0 vs. 16.6 months; HR, 0.47; 95% CI, 0.32 to 0.70)[16]. Combination olaparib plus bevacizumab treatment received approval from the Food and Drug Administration (FDA) on May 8, 2020 as a therapeutic option for patients diagnosed with HRD-positiveadvanced epithelial ovarian, fallopian tube, or primary peritoneal carcinoma that exhibited partial or complete responses to first-line platinum-based chemotherapeutic regimens [17].

While these trial efficacy data are extremely promising the relative clinical benefits and economic value of olaparib plus bevacizumab must be taken into consideration to ensure the appropriate allocation of medical resources, emphasizing the importance of conducting health economic evaluating the cost-effectiveness of this therapeutic regimen in AOC patients. By leveraging early biomarker-based patient selection strategies in light of the results of cost-effectiveness analyses, oncologists can ensure that patients recieve the most appropriate interventions on an individualized basis. As such, this study was conducted to evaluate the relative costs and efficacy outcomes associated with olaparib plus bevacizumab relative to bevacizumab alone as a first-line maintenance treatment option for AOC patients in particular molecular status subgroups.

## Materials and methods

### Clinical Data Inputs

A Markov model was designed using a hypothetical population of 806 patients with AOC based on the baseline data from patients enrolled in the PAOLA-1 trial [15, 18]. These included 537 and 269 patients that were randomly assigned to olaparib plus bevacizumab and single-agent bevacizumab treatment groups, respectively, including HRD-positive patients [n = 255 (47.5%) and n = 132 (49.1%), respectively], BRCA mutations patients [n = 161 (30.0%) and n = 80 (29.7%), respectively], and were HRD-positive but BRCA mutation-negative patients [n = 97 (18.1%) and n = 55 (20.5%), respectively][15, 16]. Per the PAOLA-1 trial protocols, enrolled patients received twice-daily oral olaparib (300 mg) treatment for a maximum 2 years, while bevacizumab was intravenously administered (15 mg/Kg) every 3 weeks for a maximum 15 months [15, 16]. These patients were assumed to have an of 60 years, with an average body weight and body surface area of 70 kg and 1.84 m^2^, respectively, and average serum creatinine levels of 1 mg/dL [15, 16, 19, 20] (Table 1). Computed tomography (CT) imaging studies of these patients were conducted every 24 weeks to detect progressive disease and to evaluate patient status [15, 16]. Patients underwent treatment with their assigned first-line maintenance therapies until developing progressive disease (PD) or experiencing adverse events (AEs) that were considered unacceptable, at which time 260 (48.4%) pateins in the combination group and 164 (61.0%) pateins in the bevacizumab single-agent group were treated using carboplatin plus paclitaxel in line with the guidelines of the PAOLA-1 trial (Table 1)[21, 22]. All other patients received best supportive care (BSC) until death, with terminal care having been provided to patients prior to death. See eTable 2 of supplementary materials further information regarding drug dosing, drug pricing, and methods of administration. The CHEERS guidelines were used to conduct this study (Supplementary Materials eTable 1).

**Table 1:** Model Parameters: Key Clinical and Health Preference Data

Parameters	Baseline value	Range		Reference	Distribution
		Minimum	Maximum		
**Clinical data**					
**Weibull survival model for OS of olaparib plus bevacizumab**					
Overall patients Patients with a tumor BRCA mutation Patients with HRD tumors Patients with HRD tumors without a BRCA mutation	Scale = 0.0032233, Shape = 1.3412093 Scale = 0.0008312, Shape = 1.4656368 Scale = 0.0013868, Shape = 1.4043253 Scale = 0.0017414, Shape = 1.4345009	- - - -	- - - -	(15)	- - - -
**Weibull survival model for PFS of olaparib plus bevacizumab**					
Overall patients Patients with a tumor BRCA mutation Patients with HRD tumors Patients with HRD tumors without a BRCA mutation	Scale = 0.03432, Shape = 0.900619 Scale = 0.005312, Shape = 1.20865 Scale = 0.014599, Shape = 0.014599 Scale = 0.025081, Shape = 0.913035	- - - -	- - - -	(16)	- - - -
**Weibull survival model for OS of bevacizumab**					
Overall patients Patients with a tumor BRCA mutation Patients with HRD tumors Patients with HRD tumors without a BRCA mutation	Scale = 0.0025282, Shape = 1.427602 Scale = 0.0016336, Shape = 1.4217268 Scale = 0.0014855, Shape = 1.500712 Scale = 0.0009159, Shape = 1.6748696	- - - -	- - - -	(15)	- - - -
**Weibull survival model for PFS of bevacizumab**					
Overall patients Patients with a tumor BRCA mutation Patients with HRD tumors Patients with HRD tumors without a BRCA mutation	Scale = 0.041625, Shape = 0.984316 Scale = 0.022464, Shape = 1.082455 Scale = 0.021614, Shape = 0.021614 Scale = 0.033704, Shape = 0.913035	- - - -	- - - -	(16)	- - - -
**Risk for main AEs in olaparib plus bevacizumab group**					
Risk of fatigue	0.050	0.040	0.060	(15, 16)	Beta
Risk of neutropenia	0.060	0.048	0.072	(15, 16)	Beta
Risk of lymphopenia	0.070	0.056	0.084	(15, 16)	Beta
Risk of anemia	0.170	0.136	0.204	(15, 16)	Beta
Risk of hypertension	0.190	0.152	0.228	(15, 16)	Beta
**Risk for main AEs in bevacizumab group**					
Risk of hypertension	0.300	0.240	0.360	(15, 16)	Beta
**Proportion of receiving active second-line treatment**					
Olaparib plus bevacizumab	0.484	0.387	0.581	(21)	Beta
Bevacizumab	0.610	0.488	0.732	(21)	Beta
**Utility and disutility**					
Utility of PFS	0.840	0.672	1.008	(25, 26)	Beta
Utility of PD	0.790	0.632	0.948	(25, 26)	Beta
Disutility of leukopenia	0.090	0.072	0.108	(23)	Beta
Disutility of fatigue	0.170	0.136	0.204	(24)	Beta
Disutility of neutropenia	0	-	-	(24)	-
Disutility of anemia	0	-	-	(24)	-
Disutility of hypertension	0	-	-	(24)	-
**Body weight (kilogram)**	70	56	84	(19, 20)	Normal
**Body surface area (meters** ^**2**^ **)**	1.84	1.47	2.21	(19, 20)	Normal
**Discount rate**	0.03	0	0.05	(24)	Uniform

Abbreviation: OS, overall survival; BRCA, breast cancer susceptibility genes; HRD, homologous recombination deficiency; PFS, progression-free survival; PD, progressed disease; AEs, adverse events

### Model Development

The cost-effectiveness of providing AOC patients with first-line maintenance therapy consisting of olaparib plus bevacizumab relative to bevacizumab alone was assessed from a US healthcare system perspective using TreeAge Software (TreeAge Pro 2021®, available at: https://www.treeage.com). The developed state-transition model included data pertaining to efficacy outcomes and total costs for hypothetical groups of AOC patient, who progressed through PFS, PD, and death as mutually exclusive health states. After beginning in the PFS state upon model initiation, each patient had a chance to transition to the PD or death state 3-week model cycle state. (Supplementary Materials eFigure 1). The model had an overall 15-year time horizon as 99% of patients were expected to be deceased according to follow-up and available survival data.

PAOLA-1 trial data were used to estimate transition probability values. Given that specific patient baseline data from this trial were not accessible, GetData Graph Digitizer (Version 2.26, available at: http://www.getdata-graph-digitizer.com/index.php) was utilized to extract survival data from published Kaplan-Meier (KM) curves. These data were then fitted with the Exponential, Log-logistic, Log-normal, Gompertz, and Weibull distributions with model fit being evaluated based on estimated values from Akaike information criterion (AIC) and Bayesian information criterion (BIC) (Supplementary Materials eFigure 2 and eTable 3). This approach ultimately revealed that individual patient data most closely conformed to the Weibull distribution. The distributions for the the γ (scale) and λ (shape) parameters were computed with R (version 4.1.1, available at: http://www.rproject.org) [19] (Table 1).

Model outcomes included overall costs, life years (LYs), QALYs, and incremental cost-effectiveness ratio (ICER) values. The willingness-to-pay (WTP) threshold when evaluating these model outcomes from a US payer’s perspective was $150,000/QALY [19, 23]. An annual discounting rate of 3% for was applied to all healthcare costs and benefits in these analyses [24].

### Utility and cost inputs

Utility values serve as means of quantifying the preference of a given patient for living in a specific health state, with values ranging from 1 (perfect health) to 0 (health). These values provide an effective means of measuring the effects of disease-associated health state on particular outcome data. As Quality of Life Questionnaire (EORTC QLQ-C30), details were not reported for the PAOLA-1 trial, the PFS and PD states were herein assigned respective average utilities of 0.84 and 0.79, as per a previous publication [25, 26]. These analyses also took the disutility values of Grade 3–4 treatment-related AEs with ≥ 5% incidence into consideration [23, 24].

Direct medical costs evaluated in these analyses included the medication costs as well as costs associated with drug administration, laboratory tests, tumor imaging, laboratory tests, testing for HRD status, testing for germline BRCA status, AE-related treatments, BSC, and terminal care (Table 2). The Centers for Medicare & Medicaid Services and drug price inquiries were used to establish medication costs [27, 28], while published data were used to determine all other costs [24, 29–31]. Grade 3–4 treatment-related AEs with a disutility value ≥ 5% were additionally taken into consideration [32].

**Table 2:** Cost Estimates (US $)

Parameters	Baseline value	Range		Reference	Distribution
		Minimum	Maximum		
**Drug cost, $/per cycle**					
Olaparib	3,657	2,926	4,388	(27)	Gamma
Bevacizumab	7,326	5,861	8,791	(28)	Gamma
Carboplatin	23	18	28	(28)	Gamma
Paclitaxel	35	28	42	(28)	Gamma
**Cost of AEs**					
Bevacizumab	76	61	91	(24)	Gamma
Olaparib plus bevacizumab	291	233	349	(24, 29)	Gamma
**Laboratory per cycle**	4	3	5	(27)	Gamma
**Tumor imaging per cycle**	105	84	126	(24)	Gamma
**Administration per cycle**	124	99	149	(24)	Gamma
**Germline BRCA testing per patient**	2,901	2,321	3,481	(30)	Gamma
**HRD test per patient**	4,682	3,746	5,618	(30)	Gamma
**Best supportive care per cycle**	4,143	3,314	4,972	(31)	Gamma
**Terminal care per patient**	85,904	68,723	103,085	(24)	Gamma

Abbreviation: AEs, adverse events; BRCA, breast cancer susceptibility genes; HRD, homologous recombination deficiency

### Sensitivity analyses

Model stability was evaluated through a series of sensitivity analyses. In one-way sensitivity analyses, each model parameter was modulated to ± 20% of the baseline in order to measure the effects of these variables on model outcomes. These results were presented using Tornado diagrams [23]. In two-way sensitivity analyses, the effects of simultaneously changing PFS utility values and other parameters on model outcomes were assessed, as one-way sensitivity analyses revealed that PFS utility values strongly influenced ICER values. In the probabilistic sensitivity analyses, 10,000 Monte Carlo simulations in which all major parameters were randomly varied within the defined distribution ranges were conducted [23]. The resultant data of this analysis were presented in the form of scatter plots and acceptability curves.

## Results

### Base-case analysis

Over the 15-year model interval, olaparib plus bevacizumab was associated with improved health outcomes and higher costs relative to single-agent bevacizumab treatment. Specifically, this combination regimen yielded 7.08, 8.92, 7.62, and 6.13 QALYs (8.42, 10.89, 9.36, and 7.52 LYs) for overall, BRCA mutations, HRD-positive, and HRD-positive without BRCA mutations AOC patients, respectively, while bevacizumab monotherapy yielded 5.23, 5.58, 5.91, and 5.16 QALYs (6.46, 6.96, 7.32, and 6.38 LYs) in these same patient cohorts. The cost of single-agent bevacizumab was calculated to be $329,087, $440,453, $374,452, and $332,850, while the olaparib plus bevacizumab treatment was $622,743, $706,121, $617,198, and $526,642 for these same patient cohorts. Relative to bevacizumab alone, olaparib plus bevacizumab treatment was thus associated with ICER values of were $158,729 ($136,218), $79,434 ($66,120), $141,636 ($117,747), and $200,595 ($169,733) per QALY (LY) in these four respective patient groups (Table 3). These results thus suggest that olaparib plus bevacizumab represents an optimal first-line maintenance therapy option for BRCA mutations or HRD-positive AOC patients.

**Table 3:** Results of the Base-Case Analysis

Treatment	Total cost $	LYs	ICER $/LY ^a^	QALYs	ICER $/QALY ^b^
**Overall Patients**					
Bevacizumab	329,087	6.46	NA	5.23	NA
Olaparib plus bevacizumab	622,743	8.42	136,218	7.08	158,729
**Patients with BRCA Mutations**					
Bevacizumab	440,453	6.96	NA	5.58	NA
Olaparib plus bevacizumab	706,121	10.89	66,120	8.92	79,434
**Patients with HRD positive**					
Bevacizumab	374,452	7.32	NA	5.91	NA
Olaparib plus bevacizumab	617,198	9.36	117,747	7.62	141,636
**Patients with HRD positive without BRCA Mutations**					
Bevacizumab	332,850	6.38	NA	5.16	NA
Olaparib plus bevacizumab	526,642	7.52	169,733	6.13	200,595

^a^ Compared to olaparib plus bevacizumab ($/LY).

^b^ Compared to olaparib plus bevacizumab ($/QALY).

Abbreviation: ICER, incremental cost-effectiveness ratio; LY, life-year; QALY, quality-adjusted life-year; BRCA, breast cancer susceptibility genes; HRD, homologous recombination deficiency

### Sensitivity analyses

**Fig. 1:**
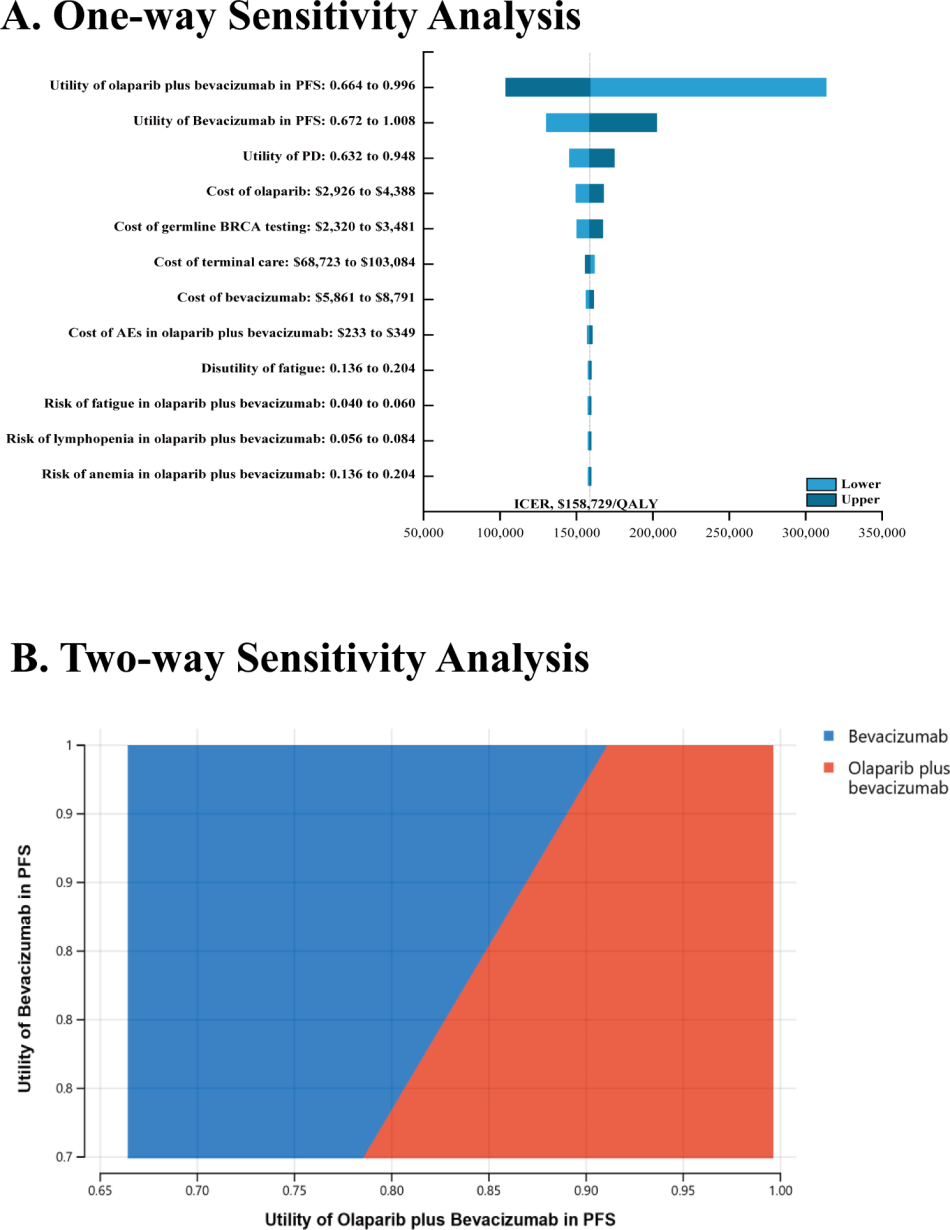
The sensitivity analyses. Abbreviation: PFS, progression-free survival; PD, progressive disease; HRD, homologous recombination deficiency; BRCA, breast cancer susceptibility genes; WTP, willingness-to-pay; QALY, quality-adjusted life-year. **Note-**Orange background indicates that ICER for olaparib plus bevacizumab versus bevacizumab are lower than WTP, while blue represents that ICER are higher than WTP

One-way sensitivity analyses indicated that the utility of the PFS in olaparib plus bevacizumab (varying from 0.664 to 0.996, yielding ICERs from $104,047/QALY to $313,189/QALY) and the utility of the PFS in olaparib plus bevacizumab (varying from 0.672 to 1.008, yielding ICERs from $130,588/QALY to $202,330/QALY) most significantly affected ICERs values generated by this model. In contrast, the cost of follow-up costs, the cost of second-line treatment costs, and the cost of AE-related treatment costs in the bevacizumab group largely failed toimpact model results (Fig. 1A). Two-way sensitivity analyses demonstrated that when the utility of PFS in the olaparib plus bevacizumab patients group was higher than 0.913 and the utility value for the bevacizumab was varied within the specified range, the olaparib plus bevacizumab will be a cost-effective treatment option (Fig. 1B).

**Fig. 2:**
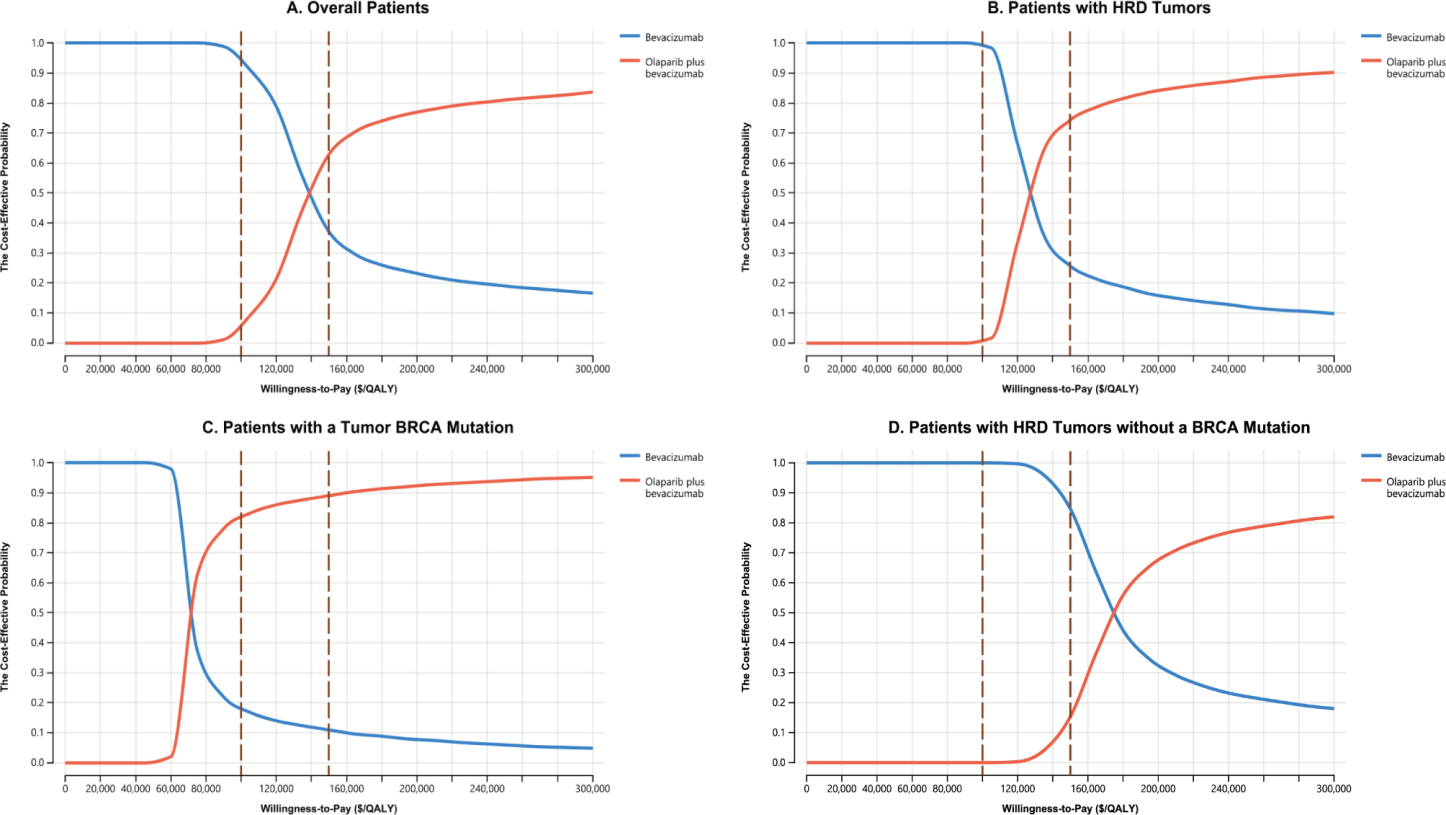
The cost-effectiveness acceptability curves for olaparib plus bevacizumab strategy compared to bevacizumab strategy in the overall patients (**A**), Patients with HRD tumors (**B**), Patients with a tumor BRCA mutation (**C**), and Patients with HRD tumors without a BRCA mutation (**D**). Abbreviation: QALY, quality-adjusted life-year.

Acceptability curves generated based on the results of probabilistic sensitivity analyses revealed that increasing the WTP threshold resulted in higher odds of combination olaparib plus bevacizumab maintenance therapy being cost-effective, with a 50% chance of this combination regimen being cost-effective relative to bevacizumab alone at WTP thresholds of $138,000 and $175,000 per QALY, there was a 50% chance that olaparib plus bevacizumab was cost-effective compared with bevacizumab alone for overall AOC patients and HRD-positive without BRCA mutation-negative AOC patients, respectively (Fig. 2). At a US WTP threshold of $150,000/QALY, these analyses suggested that the overall odds of olaparib + bevacizumab being cost-effective relative to single-agent bevacizumab treatment being cost-effective in the overall, BRCA mutation-positive, HRD-positive, and HRD-positive BRCA mutation-negative AOC patient populations were 63.3%, 89.1%, 74.3%, and 15.3% (Fig. 2 and Supplementary Materials eFigure 3).

## Discussion

Healthcare costs associated with OC estimated at $6.4 billion in the USA in 2020, and respective predicted increases in national medical service and prescription drug costs of 34% and 17% as of 2030[33, 34]. Given the continuously increasing costs of healthcare, there is a pressing need for value-based oncological care. The development of olaparib and related PARP inhibitors including niraparib and rucaparib has rapidly transformed the treatment landscape for AOC patients. In the SOLO1 trial demonstrated that maintenance olaparib monotherapy was associated with significant clinical improvements in newly diagnosed AOC, yielding a median PFS of 56 months in treated individuals [35]. In their analyses, Muston et al. found that first-line maintenance olaparib treatment yielded an ICER of $51,986/QALY, such that it was a cost-effective alternative to routine monitoring in AOC patients [36]. Other studies reported that combination of PARP inhibitors with anti-angiogenic agents yielded superior PFS outcomes relative to PARP inhibitor treatment alone [18, 21, 37–39]. The final OS and PFS results from the PAOLA-1 trial provided strong support for the benefits of first-line maintenance treatment with olaparib plus bevacizumab for patients with AOC. In light of these new data, there is a need for the publication of revised calculations pertaining to the cost-effectiveness of this combined treatment regimen. As such, this study was conducted with the goal of comparing the cost-effectiveness of olaparib plus bevacizumab to that of bevacizumab monotherapy as a first-line maintenance treatment for patients with AOC in particular clinical subgroups from the perspective of the US healthcare sector.

The decision analysis model developed herein indicated that olaparib plus bevacizumab was not a cost-effective alternative to single-agent bevacizumabtreatment in the overall AOC population, with an ICER of $158,729/QALY exceeding the selected US WTP threshold of $150,000/QALY, The incremental costs of this combination treatment regimen mainly resulted from drug costs and costs associated with the management cost of treatment-related AEs, suggesting that efforts to lower treatment costs and to prevent AE incidence may contribute to improved cost-effectiveness. In one-way sensitivity analyses, combined olaparib + bevacizumab was found to be cost-effective if the PFS utility value for the olaparib + bevacizumab group was greater than 0.845 and that for the bevacizumab group was below 0.795. In light of these data, additional two-way sensitivity analyses were performed in which the $150,000/QALY threshold was used to assess the cost-effectiveness of olaparib + bevacizumab for different utility value combinations. In probabilistic sensitivity analyses the odds of olaparib plus bevacizumab being cost-effective were 63.3% relative to single-agent bevacizumab. Data from the PAOLA-1 trial suggested that the OS of patients administered this combination regimen only extended by a non-significant 4.9 months [15]. Given the higher costs of combination treatment and this absence of significant clinical efficacy, this likely explains the finding that olaparib plus bevacizumab was not an economical alternativeto bevacizumab monotherapy. Future pricing adjustments will thus be essential to achieve a greater balance between the costs and benefits associated with this first-line maintenance regimen such that it will have the potential for broader clinical application.

While PARP inhibitor therapy has conferred survival and QoL benefits to many patients diagnosed with OC, not all patients respond to such treatment, underscoring a need to identify the patients who are best suited for PARP inhibitor administration. Biomarker testing strategies offer a means of selecting treatment regimens on an individualized basis. As such, the BRCA mutation status and HRD status of AOC patients were taken into consideration in the present study in an effort to provide better evidence-based guidance for both healthcare providers and payers. The calculated ICERs of olaparib plus bevacizumab versus bevacizumab monotherapy were $79,434/QALY, $141,636/QALY, and $ 200,595/QALY for BRCA mutations, HRD-positive, and HRD-positive without BRCA mutations AOC patients, respectively, suggesting that this combined treatment regimen is only cost-effective for the former two patient subgroups in line with prior evidence [40–43]. Two recent retrospective reports focused on 33 and 42 AOC patients in France and China with BRCA mutations or HRD-positive disease, respectively, found that PARP inhibitor treatment was associated with, the median PFS and OS in BRCA mutation-positive patients of 20.9 vs. 37.7 months (P = 0.21) and 151.2 vs. 122.5 months (P = 0.52), whereas HRD status was identified as an independent predictor of PFS (HR, 0.67; 95%CI, 0.49 to 0.92; P = 0.01)[40, 41]. In two other meta-analyses enrolling 5,005 and 3,070 OC patients. PARP inhibitor treatment was associated with significantly improved PFS in both BRCA mutations (HR, 0.29; 95%CI, 0.24 to 0.34 and 0.34; 0.28 to 0.41) and HRD-positive (0.40; 0.32 to 0.48 and 0.39; 0.29 to 0.53) OC patients [42, 43]. Given the high costs associated with these novel therapeutic regimens, alternative treatment options for AOC patients should be taken into consideration in light of their molecular status, and the evaluation of these prognostic biomarkers at an early time point remains essential to ensuring that these patients experience optimal.

There are several key strengths to this study. First, these results are based on the most up-to-date PAOLA-1 trial results, including final OS/PFS, QoL, molecular status-related data published from 2023[16, 44]. As this trial included long-term follow-up outcome data, the models developed in the present study are more robust. In addition, all medical costs were performed after adjusting prices based on the most recent data available for the USA from 2022, ensuring that the effects of variable medical costs on study results would be minimal. Finally, these analyses were performed for both the overall cohort of AOC patients as well as for patients in three specific molecular subgroups, yielding data that may ultimately guide real-world clinical decision-making.

These results are subject to certain limitations. For one, these analyses were conducted solely based on the survival data derived from the phase III PAOLA-1 trial given that it is the only clinical comparison of the safety and efficacy of bevacizumab with or without olaparib as first-line maintenance therapy for AOC patients in different molecular status-based subgroups. Any biases stemming from the design of the PAOLA-1 trial will thus have an impact on the results of this study. only AEs of grade 3 or above were considered in the present calculations given that grade 1–2 AEs generally have a less significant effect on patients. While this may have contributed to the underestimation of AE-related costs to some degree, this factor failed to affect the results of base-case analyses in one-way sensitivity analyses, suggesting that this effect was minimal. Moreover, second-line treatment and BSC were assumed to be administered to all patients upon disease progression without considering the potential for continued primary treatment in the PD state given that no guidelines or evidence related to the latter possibility were available. However, one-way sensitivity analyses also suggested that second-line treatment did not have a major impact on model outcomes. The PAOLA-1 study was also a multicenter trial enrolling patients of varying ethnicities from multiple countries, and treatment plans for these patients were adjusted on an individualized basis, particularly over the course of follow-up. Further clinical trials will thus be necessary for more granular analyses of particular patient populations, follow-up regimens, and other variables that may affect the present results.

## Conclusion

In conclusion, these findings based on the most recent PAOLA-1 trial PFS and OS results suggest that, at current pricing levels, olaparib plus bevacizumab is not cost-effective as compared single-agent bevacizumab treatment as a first-line maintenance therapy for AOC patients. However, this regimen may be cost-effective in subsets of AOC patients who are BRCA mutations and HDR-positive. Early biomarker to identify patients in these molecular status subgroups may thus provide an opportunity to more effectively select patients that are likely to derive benefits from this combined maintenance therapy regimen, ensuring that these patients receive the most cost-effective treatment options available.

### Electronic supplementary material

Below is the link to the electronic supplementary material.

**Supplementary file 1: eFigure 1**. Model Structure. **eFigure 2**. Kaplan-Meier Curve Fitting and Extrapolation. eFigure 3. Probability Sensitivity Analysis Scatter Plot. **eTable 1.** CHEERS Checklist. **eTable 2**. Drug Dose and Cost. **eTable 3**. Summary of Statistical Goodness-of-fit of K-M Curve

## Data Availability

All authors had full access to all of the data in this study and take complete responsibility for the integrity of the data and accuracy of the data analysis. The datasets generated and/or analyzed during the current study are available from the corresponding author upon reasonable request.
