# Supplementary material for: The cost-effectiveness analysis of maintenance olaparib plus Bevacizumab in patients with advanced ovarian cancer: based on the final survival results from PAOLA-1 trial
Source: J Ovarian Res. 2023 Aug 21;16:168. doi: 10.1186/s13048-023-01257-4 (PMC10441725; doi:10.1186/s13048-023-01257-4)
Supplement: Supplementary file 1 — eFigure 1. Model Structure. eFigure 2. Kaplan-Meier Curve Fitting and Extrapolation. eFigure 3. Probability Sensitivity Analysis Scatter Plot. eTable 1. CHEERS Checklist. eTable 2. Drug Dose and Cost. eTable 3. Summary of Statistical Goodness-of-fit of K-M Curve [file 13048_2023_1257_MOESM1_ESM.pdf]

eFigure 1. Model Structure.

eFigure 2. Kaplan-Meier Curve Fitting and Extrapolation.

eFigure 3. Probability Sensitivity Analysis Scatter Plot.

eTable 1. CHEERS Checklist.

eTable 2. Drug Dose and Cost.

eTable 3. Summary of Statistical Goodness-of-fit of K-M Curve.

eFigure 1. Model Structure.

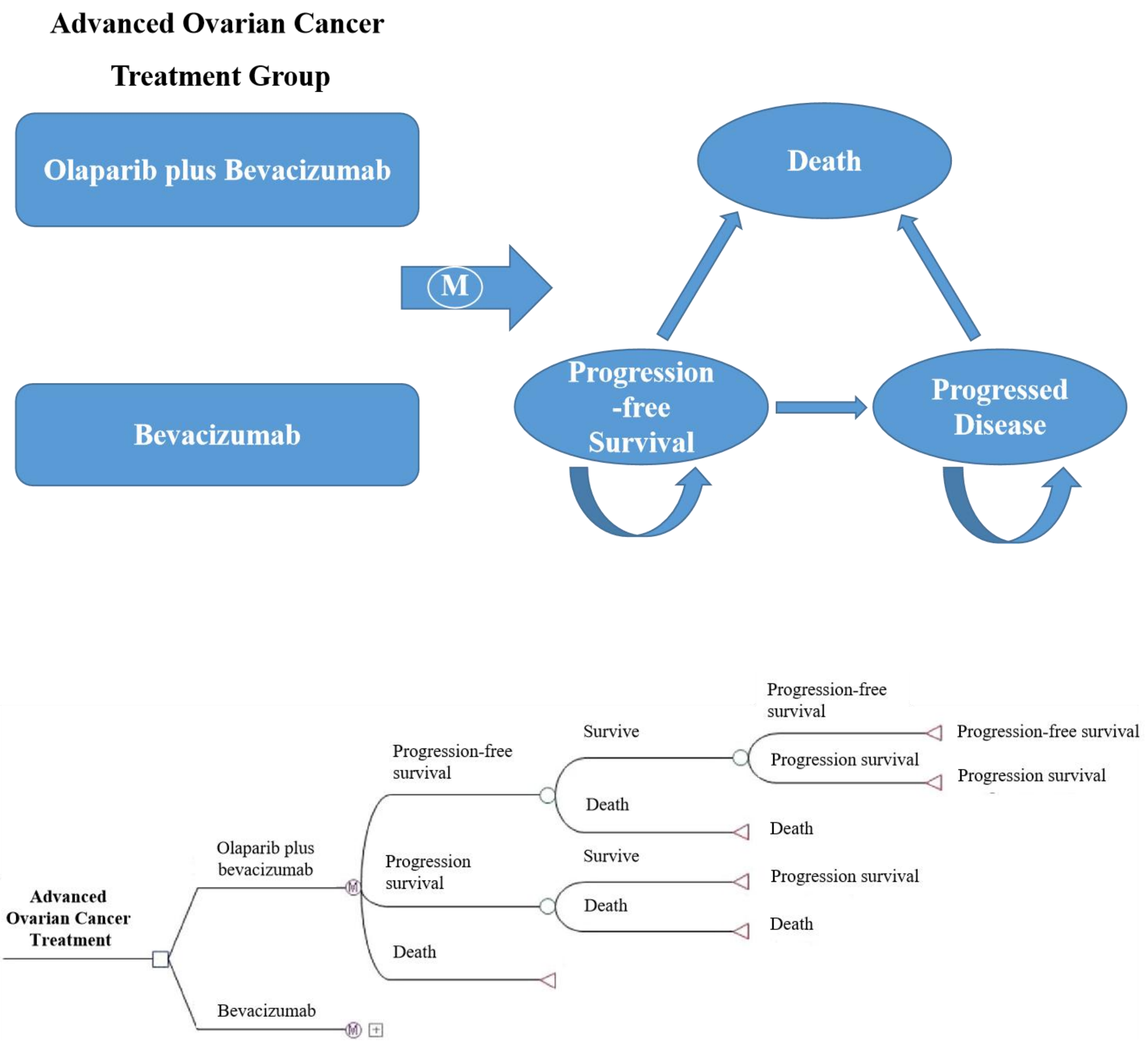

Abbreviation: M, Markov.

eFigure 2. Kaplan-Meier Curve Fitting and Extrapolation.

A. Overall Survival among All Patients

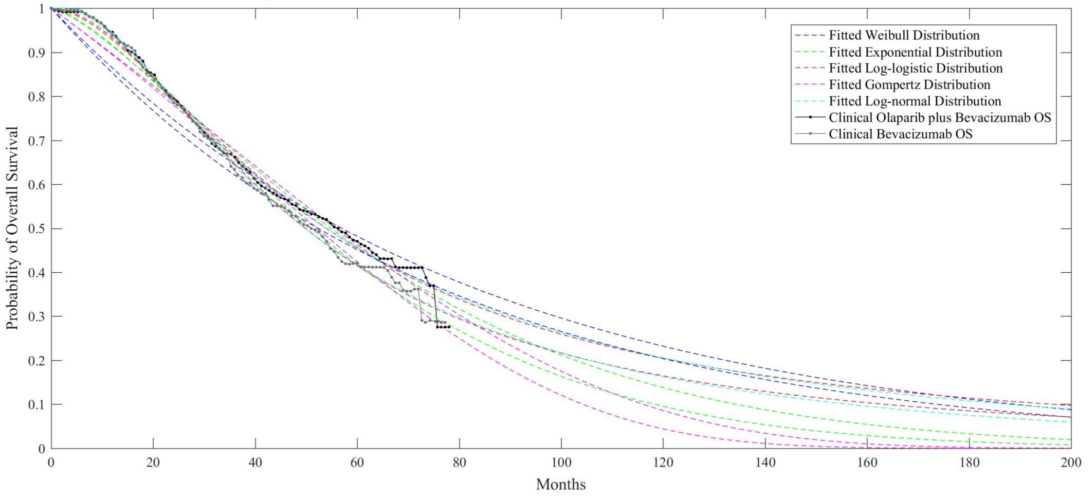

B. Progression-free survival among All Patients

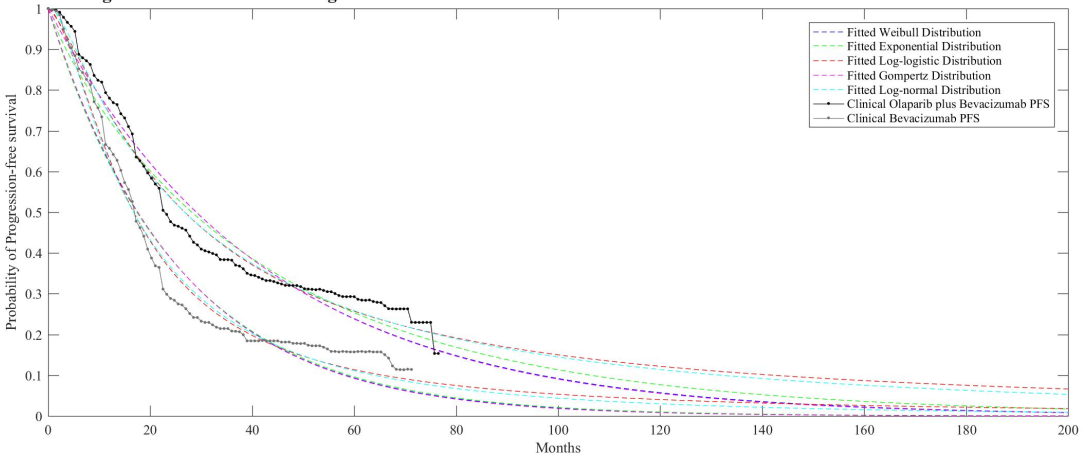

C. Overall Survival in Patients with a BRCA mutation

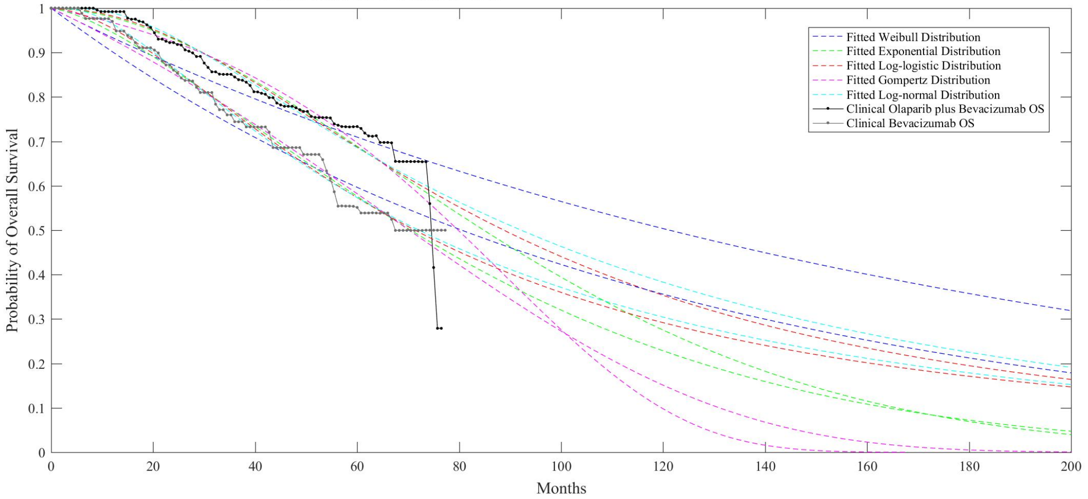

D. Progression-free Survival in Patients with a BRCA mutation

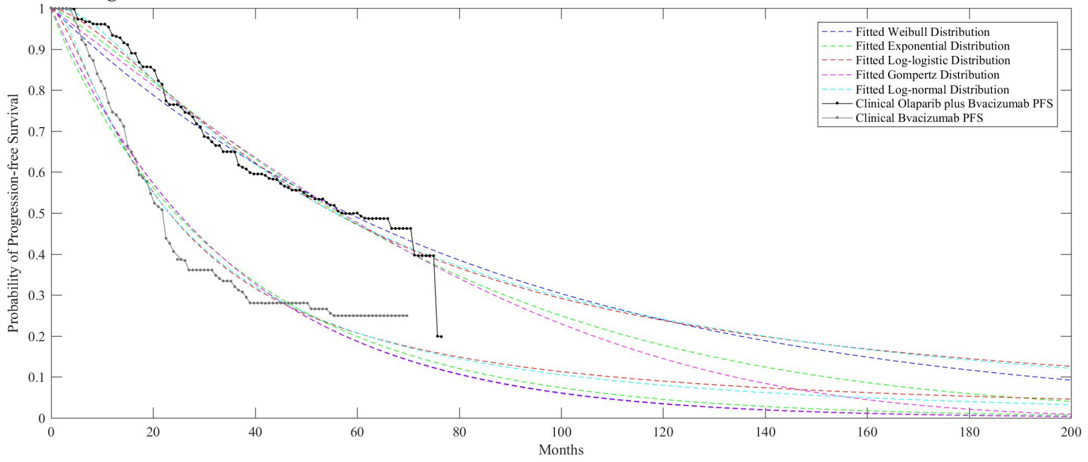

E. Overall Survival in Patients with HRD Tumors

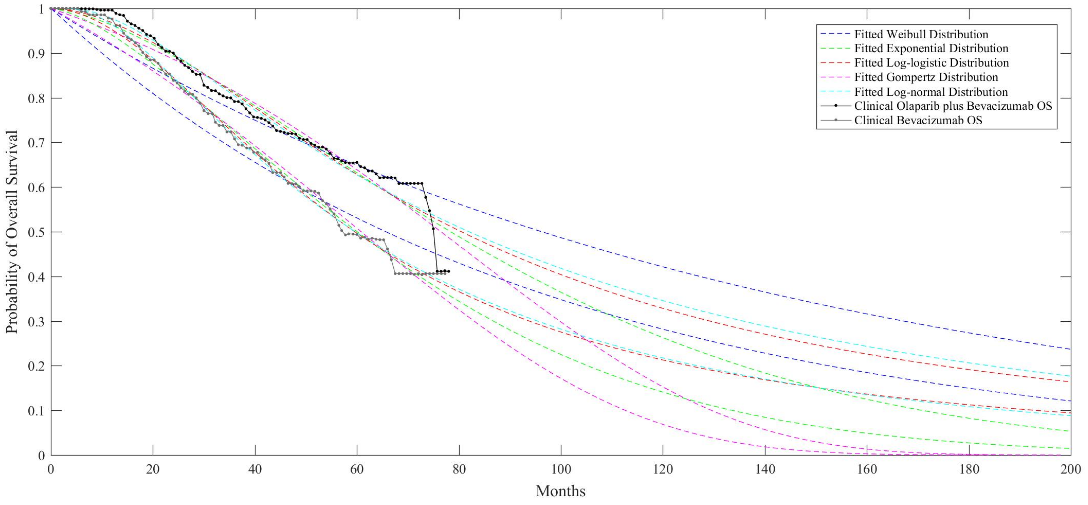

F. Progression-free Survival in Patients with HRD Tumors

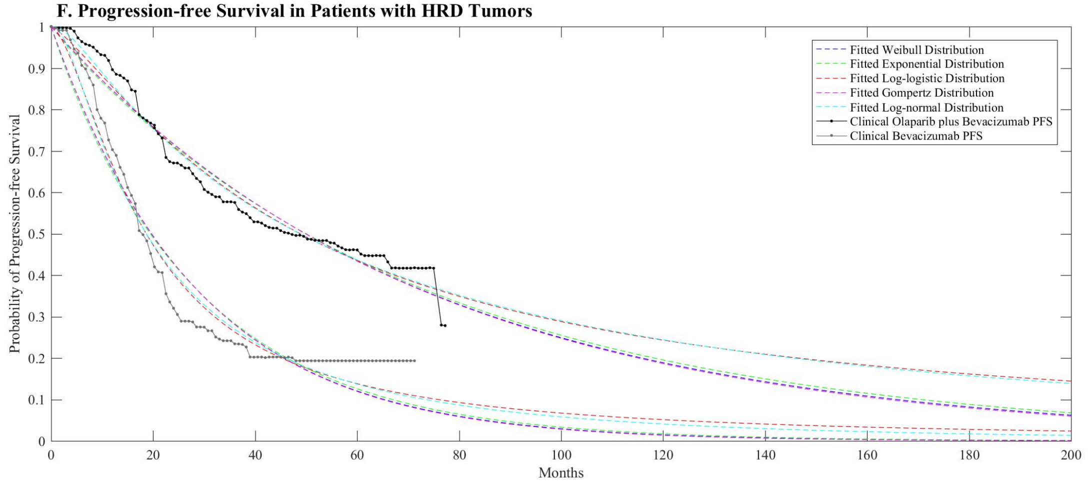

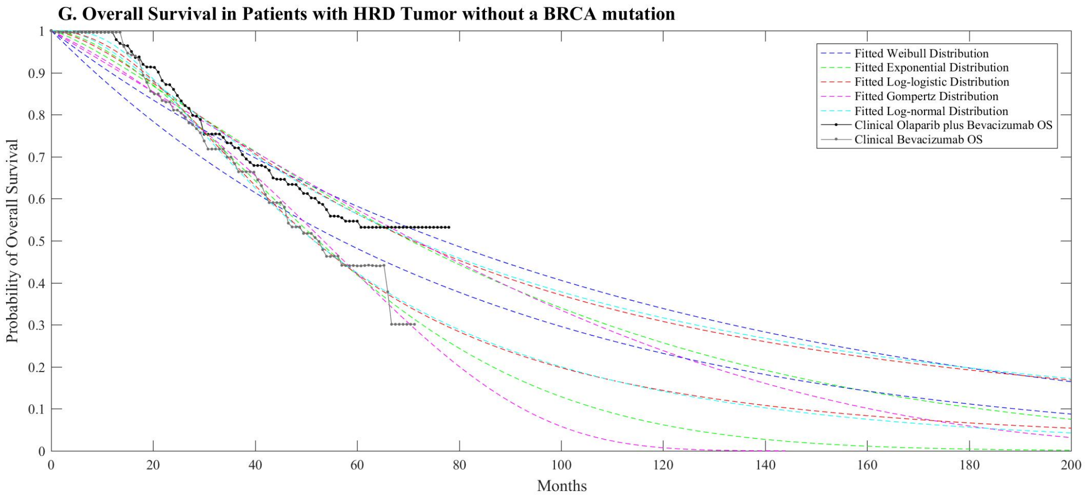

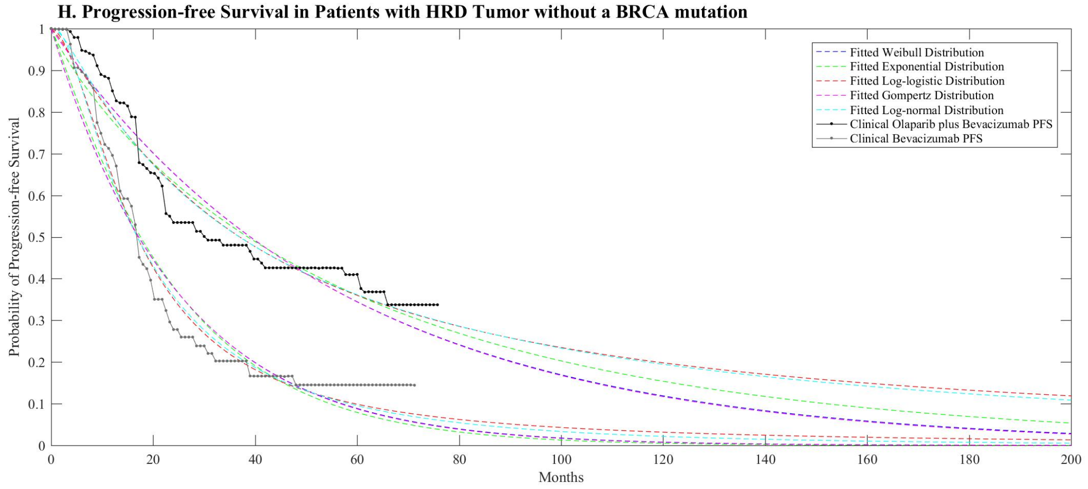

Abbreviation: OS, overall survival; PFS, progression-free survival; BRCA, breast cancer susceptibility genes; HRD, homologous recombination deficiency.

Kaplan-meier curve fitting and extrapolation for overall survival in the overall patients (A), progression-free survival in the overall patients (B), overall survival in patients with a tumor BRCA mutation (C), progression-free survival in patients with a tumor BRCA mutation (D), overall survival in patients with HRD tumors (E), progression-free survival in patients with HRD tumors (F), overall survival in patients with HRD without a BRCA mutation (G), and progression-free survival in patients with HRD without a BRCA mutation (H) respectively.

eFigure 3. Probability Sensitivity Analysis Scatter Plot.

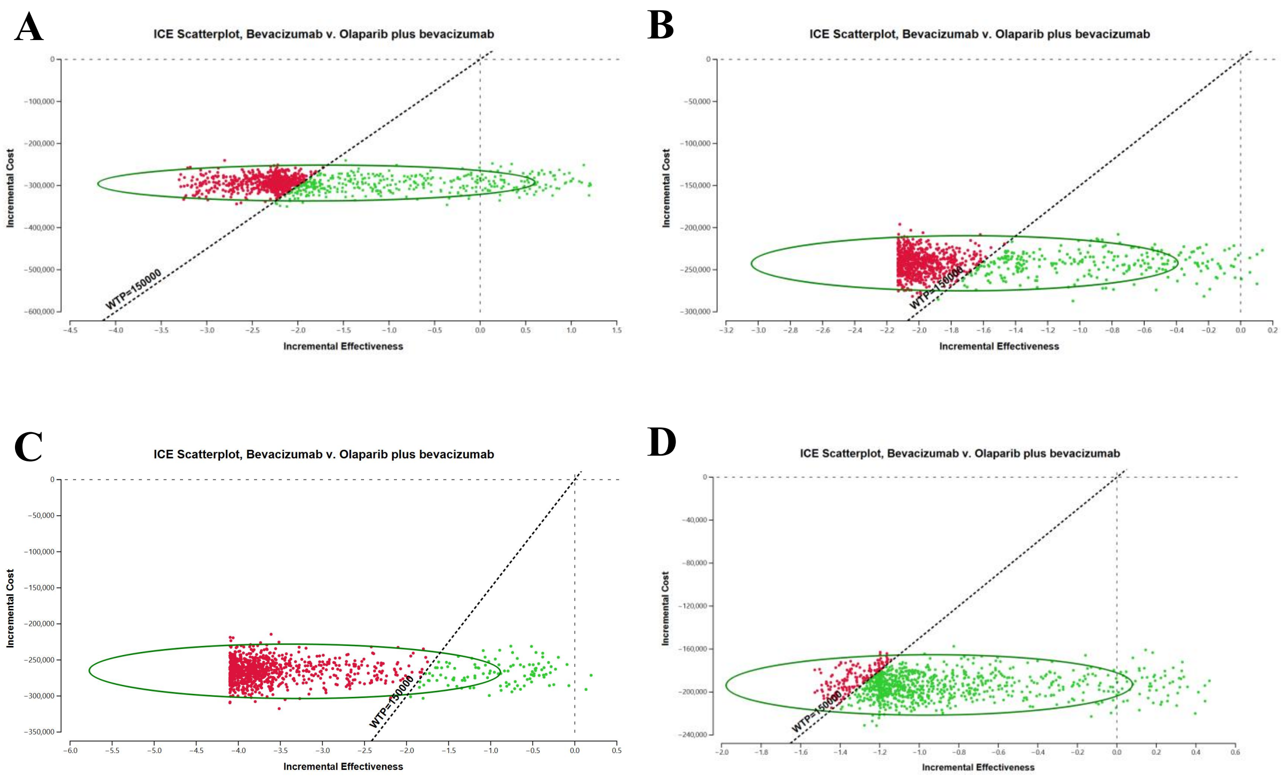

Abbreviation: WTP, willingness-to-pay.

Each point in the diagram represents a simulation result of 10,000 Monte Carlo simulation. Ellipse represent the 95% CI and dotted line represent WTP of $150,000/QALY in the overall patients (A), patients with HRD tumors (B), patients with a tumor BRCA mutation (C), and patients with HRD tumors without a BRCA mutation (D) respectively. The red dots represent the probability that olaparib plus bevacizumab will be cost-effective.

eTable 1. CHEERS Checklist.

Section/item	Item No	Recommendation	Reported on page No
**Title and abstract**			
Title	1	Identify the study as an economic evaluation or use more specific terms such as “cost-effectiveness analysis”, and describe the interventions compared.	I
Abstract	2	Provide a structured summary of objectives, perspective, setting, methods (including study design and inputs), results (including base case and uncertainty analyses), and conclusions.	IV-V
**Introduction**			
Background and objectives	3	Provide an explicit statement of the broader context for the study.	1-3
		Present the study question and its relevance for health policy or practice decisions.	
**Methods**			
Target population and subgroups	4	Describe characteristics of the base case population and subgroups analysed, including why they were chosen.	3
Setting and location	5	State relevant aspects of the system(s) in which the decision(s) need(s) to be made.	4, 5
Study perspective	6	Describe the perspective of the study and relate this to the costs being evaluated.	4, 5
Comparators	7	Describe the interventions or strategies being compared and state why they were chosen.	3
Time horizon	8	State the time horizon(s) over which costs and consequences are being evaluated and say why appropriate.	4
Discount rate	9	Report the choice of discount rate(s) used for costs and outcomes and say why appropriate.	5
Choice of health outcomes	10	Describe what outcomes were used as the measure(s) of benefit in the evaluation and their relevance for the type of analysis performed.	4, 5
Measurement of effectiveness	11a	*Single study-based estimates:* Describe fully the design features of the single effectiveness study and why the single study was a sufficient source of clinical effectiveness data.	3-5

	11b	*Synthesis-based estimates:* Describe fully the methods used for identification of included studies and synthesis of clinical effectiveness data.	
Measurement and valuation of preference based outcomes	12	If applicable, describe the population and methods used to elicit preferences for outcomes.	Not applicable
Estimating resources and costs	13a	*Single study-based economic evaluation:* Describe approaches used to estimate resource use associated with the alternative interventions. Describe primary or secondary research methods for valuing each resource item in terms of its unit cost. Describe any adjustments made to approximate to opportunity costs.	2-5
	13b	*Model-based economic evaluation:* Describe approaches and data sources used to estimate resource use associated with model health states. Describe primary or secondary research methods for valuing each resource item in terms of its unit cost. Describe any adjustments made to approximate to opportunity costs.	
Currency, price date, and conversion	14	Report the dates of the estimated resource quantities and unit costs. Describe methods for adjusting estimated unit costs to the year of reported costs if necessary. Describe methods for converting costs into a common currency base and the exchange rate.	5
Choice of model	15	Describe and give reasons for the specific type of decision-analytical model used. Providing a figure to show model structure is strongly recommended.	4<br>eFigure 1
Assumptions	16	Describe all structural or other assumptions underpinning the decision-analytical model.	3-5
Analytical methods	17	Describe all analytical methods supporting the evaluation. This could include methods for dealing with skewed, missing, or censored data; extrapolation methods; methods for pooling data; approaches to validate or make adjustments (such as half cycle corrections) to a model; and methods for handling population heterogeneity and uncertainty.	3-5<br>eFigure 2<br>eTable 5
**Results**			

Study parameters	18	Report the values, ranges, references, and, if used, probability distributions for all parameters. Report reasons or sources for distributions used to represent uncertainty where appropriate. Providing a table to show the input values is strongly recommended.	3-5<br>Table 1
Incremental costs and outcomes	19	For each intervention, report mean values for the main categories of estimated costs and outcomes of interest, as well as mean differences between the comparator groups. If applicable, report incremental cost-effectiveness ratios.	6<br>Table 2
Characterising uncertainty	20a	*Single study-based economic evaluation:* Describe the effects of sampling uncertainty for the estimated incremental cost and incremental effectiveness parameters, together with the impact of methodological assumptions (such as discount rate, study perspective).	6, 7
	20b	*Model-based economic evaluation:* Describe the effects on the results of uncertainty for all input parameters, and uncertainty related to the structure of the model and assumptions.	
Characterising heterogeneity	21	If applicable, report differences in costs, outcomes, or cost-effectiveness that can be explained by variations between subgroups of patients with different baseline characteristics or other observed variability in effects that are not reducible by more information.	Not applicable
**Discussion**			
Study findings, limitations, generalisability, and current knowledge	22	Summarise key study findings and describe how they support the conclusions reached. Discuss limitations and the generalisability of the findings and how the findings fit with current knowledge.	7-10
**Other**			
Source of funding	23	Describe how the study was funded and the role of the funder in the identification, design, conduct, and reporting of the analysis. Describe other non-monetary sources of support.	12
Conflicts of interest	24	Describe any potential for conflict of interest of study contributors in accordance with journal policy. In the absence of a journal policy, we recommend authors comply with International Committee of Medical Journal Editors recommendations.	12

A good template page for CHEERS Checklist is as follows: http://www.ispor.org/TaskForces/EconomicPubGuidelines.asp.

Reference:

Husereau D, Drummond M, Petrou S, et al. Consolidated health economic evaluation reporting standards (CHEERS) — Explanation and elaboration: A report of the ISPOR health economic evaluations publication guidelines good reporting practices task force. Value Health 2013;16:231-50.

eTable 2. Drug Dose and Cost.

Drug	Dose	Time	Unit costs ($/mg)
Olaparib plus bevacizumab	Olaparib, 300 mg	Administered orally 300 mg twice daily for a total duration of up to 2 years.	0.8717
	Bevacizumab, 15 mg/kg	Administered intravenously every 3 weeks for a total duration of up to 15 months	6.9772
Bevacizumab	Bevacizumab, 15 mg/kg	Administered intravenously every 3 weeks for a total duration of up to 15 months	6.9772
Carboplatin plus paclitaxel	Carboplatin, AUC 5	Administered intravenously every 3 weeks	0.0533
	Paclitaxel, 175 mg/m^2		0.1090

Abbreviation: AUC, area under curve.

eTable 3. Summary of Statistical Goodness-of-fit of K-M Curve.

	Exponential	Weibull	Gompertz	Log-logistic	Log-normal
**Overall patients**					
**Olaparib plus bevacizumab OS curve**					
AIC	103.8809	**101.7200**	104.3993	103.2912	102.9073
BIC	109.1889	**104.3740**	109.7072	108.5992	108.2152
**Bevacizumab OS curve**					
AIC	111.6172	**108.4782**	110.3209	110.7575	112.3131
BIC	116.9060	**111.1225**	115.6096	116.0463	117.6019
**Olaparib plus bevacizumab PFS curve**					
AIC	190.0193	**185.8349**	192.3351	187.9281	187.3639
BIC	192.6541	**190.1043**	197.6046	193.1976	192.6334
**Bevacizumab PFS curve**					
AIC	272.3728	**262.3637**	275.1582	264.5236	264.6514
BIC	277.5015	**264.9281**	280.2869	269.6523	269.7801
**Patients with a Tumor BRCA mutation**					
**Olaparib plus bevacizumab OS curve**					
AIC	97.7849	**95.9613**	98.0637	97.4483	97.1013
BIC	103.0543	**98.5960**	103.3331	102.7178	102.3708
**Bevacizumab OS curve**					
AIC	184.5300	**182.1468**	187.3794	184.0170	183.2119
BIC	189.6166	**187.6900**	192.466	189.1036	188.2985
**Olaparib plus bevacizumab PFS curv1e**					
AIC	26.8811	**26.1165**	27.1793	26.7733	26.6222
BIC	30.8216	**28.0868**	31.1199	30.7139	30.5628

**Bevacizumab PFS curve**					
AIC	78.7918	**74.0874**	78.5919	78.1585	77.7299
BIC	82.8064	**76.0947**	82.6066	82.1731	81.7446
**Patients with HRD Tumors**					
**Olaparib plus bevacizumab OS curve**					
AIC	62.3164	**62.0627**	62.7901	62.1264	61.8762
BIC	67.6433	**64.7261**	68.1169	67.4533	67.2031
**Bevacizumab OS curve**					
AIC	88.3579	**86.8182**	89.0415	87.8653	87.4652
BIC	93.6467	**89.4626**	94.3303	93.1541	92.7540
**Olaparib plus bevacizumab PFS curve**					
AIC	114.0459	**112.0700**	114.1329	113.9370	113.5641
BIC	119.3347	**114.7143**	119.4217	119.2257	118.8529
**Bevacizumab PFS curve**					
AIC	242.5558	**237.2227**	245.8001	239.3863	238.5657
BIC	247.6845	**240.7870**	250.9288	244.5150	243.6944
**Patients with HRD Tumor without a BRCA mutation**					
**Olaparib plus bevacizumab OS curve**					
AIC	77.1851	**75.8806**	77.5736	77.1359	76.7608
BIC	82.4930	**78.5346**	82.8815	82.4438	82.0687
**Bevacizumab OS curve**					
AIC	87.6670	**85.3103**	88.5524	86.9933	86.6210
BIC	92.7956	**87.8746**	93.6811	92.1220	91.7497
**Olaparib plus bevacizumab PFS curve**					
AIC	140.0794	**139.1778**	141.2162	140.1730	139.7070
BIC	145.3293	**141.8028**	146.4661	145.4229	144.9569

**Bevacizumab PFS curve**					
AIC	288.4599	**277.0903**	281.2564	277.1455	277.6555
BIC	293.5885	**280.9546**	286.3851	282.2742	282.7842

Abbreviation: OS, overall survival; PFS, progression-free survival; AIC, Akaike's information criterion; BIC, Bayesian information criterion; BRCA, breast cancer susceptibility genes; HRD, homologous recombination deficiency.

.

According to the AIC and BIC value of the distributions and the visual fitting of the curves (eFigure 2) showed that the Weibull distribution is probably the most reasonable parametric survival model. Weibull distribution is flexible and widely used for matching patients with three states over time, because it can monotonously increase or decrease risk functions, and it is suitable for estimating events occurring during early follow-up work.
